# Experimental support for the effects of a probiotic/digestive enzyme supplement on serum cholesterol concentrations and the intestinal microbiome

**DOI:** 10.1186/s12967-016-0945-2

**Published:** 2016-06-22

**Authors:** Thomas E. Ichim, Amit N. Patel, Kim A. Shafer

**Affiliations:** Institute for Molecular Medicine, Huntington Beach, CA USA; Department of Medicine, University of Utah, Salt Lake City, UT USA; Daily Body Restore, LLC, Milford, MI USA

**Keywords:** Probiotic, Digestive enzyme, Cholesterol, Low-density lipoprotein

## Abstract

**Background:**

Elevated levels of blood cholesterol are associated with cardiovascular disease, a leading cause of morbidity and mortality worldwide. Current therapies for addressing elevated blood cholesterol can be inadequate, ineffective or associated with side effects; therefore, the search for additional therapies is ongoing. This study evaluated Daily Body Restore (DBR), a proprietary blend of 9 probiotic organisms of the genera *Lactobacillus* and *Bifidobacterium*, and 10 digestive enzymes, for its effects on cholesterol metabolism using an in vitro system and a mouse model.

**Methods:**

We used a murine model of hypercholesterolemia induced by a high fat diet to evaluate the effects of DBR on blood cholesterol concentrations. Hypercholesterolemic mice were supplemented with DBR in their drinking water for 8 weeks and compared to control mice given low fat diets or unsupplemented high fat diets. To evaluate the effects of DBR on the activity of gut microbiota in vitro, the Shime^®^ system consisting of sequential colon reactors was supplemented with DBR for analysis of short chain fatty acid production.

**Results:**

Analysis of hypercholesterolemic mice after 4 and 8 weeks of DBR supplementation revealed significant decreases in blood concentrations of low-density lipoprotein (LDL) and increases in high-density lipoprotein (HDL) while triglyceride concentrations were unaltered. Specifically, after 4 weeks of DBR supplementation, there was a 47 % decrease in LDL and a 32 % increase in HDL in peripheral blood compared to unsupplemented, high fat diet-fed mice. After 8 weeks of DBR treatment, LDL concentrations were dramatically reduced by 78 % and HDL was increased by 52 % relative to control mice. Addition of DBR to the Shime^®^ system led to significantly increased production of propionate in colon reactors, indicative of microbial production of short chain fatty acids known to inhibit cholesterol synthesis.

**Conclusions:**

DBR, a probiotic and digestive enzyme supplement, lowered harmful LDL and increased HDL levels in a mouse model and also exerted in vitro effects consistent with cholesterol-lowering activity. Given the magnitude of the effects of DBR, these findings are promising for clinical implementation of DBR for treating hypercholesterolemia.

## Background

Cardiovascular disease is a leading cause of death worldwide for which several risk factors have been identified, a major one being hypercholesterolemia. A central goal in cardiovascular disease prevention and treatment is reducing the level of serum cholesterol in individuals that present with elevated serum levels of low-density lipoprotein (LDL) cholesterol and/or triglyercides. Both pharmacological and non-pharmacological interventions, including drug therapy, dietary interventions, and exercise can reduce cholesterol concentrations in serum [1]. Many patients prefer non-drug treatments for treating elevated serum cholesterol owing to side effects of anti-lipid drugs, contraindications for use of these medications, or personal preferences. However, nondrug options involving lifestyle and dietary modifications are not always effective; therefore, natural means for addressing hyperlipidemia have been investigated experimentally and clinically, including nutraceuticals, functional foods and other dietary supplements.

Probiotics are living microorganisms which when administered in adequate amounts confer upon a health benefit upon the host. Pioneering studies in the 1970s demonstrated that fermented milk containing a *Lactobacillus* strain exerted cholesterol-lowering effects in humans [2]. Numerous studies have since confirmed that some of the lactic acid bacteria, mainly *Lactobacillus* and *Bifidobacteria* strains, are capable of modulating hypercholesterolemia [3, 4]. In vitro studies and animal models have also been used to determine the effects of probiotic interventions on metabolic processes and gene expression related to cholesterol regulation. Studies of the mechanisms by which probiotics lower cholesterol levels have revealed several affected pathways. It has been demonstrated that lactic acid bacteria ferment food-derived indigestible carbohydrates to produce short-chain fatty acids in the gut, which can then cause a decrease in the systemic levels of blood lipids by inhibiting hepatic cholesterol synthesis and/or by redistributing cholesterol from plasma to the liver [5]. Additionally, probiotic bacteria are also capable of directly assimilating cholesterol and interfering with cholesterol absorption from the gut. On this basis, there exists a strong rationale for daily consumption of probiotic products as one strategy for addressing hypercholesterolemia in patients at-risk or afflicted with cardiovascular disease.

Numerous probiotic species and combinations thereof have been employed as supplements for treating hypercholesterolemia as well as for a breadth of disorders of metabolism and digestion [6]. Unfortunately, in the field of probiotic research, there has been a mixture of both positive effects and contradictory results derived from human studies [7–13]. A recent meta-analysis demonstrated that long-term (>4 week) probiotic interventions can effectively reduce serum LDL and triglycerides in a statistically significant manner [14]. While the prospects for using probiotics to treat hypercholesterolemia are promising, information about which probiotic species or combinations thereof are actually effective, dosing regimens, and formulation protocols is lacking. The point must be raised that different probiotic preparations are anticipated to have variable cholesterol-lowering activities. Indeed, each bacterial species is unique, having distinct biological effects, and therefore, distinct health-promoting effects when used individually or in combination with other species [6]. Selection of the appropriate probiotic strains for development of supplements and food products must take into account their viability and metabolic activity during the manufacturing process, in the specific delivery vehicle, as well as in the gut microenvironment [15, 16].

This study evaluated Daily Body Restore (DBR), a proprietary blend of 9 probiotic organisms of the genera *Lactobacillus* and *Bifidobacterium*, as well as 10 digestive enzymes, for its efficacy at modifying blood cholesterol levels. We utilized a mouse model of diet-induced hypercholesterolemia and an in vitro system, the Simulator of Human Intestinal Microbial Ecosystem (Shime^®^), where the influence of DBR on fermentation by intestinal microbiota was tested. These studies not only demonstrate the cholesterol-lowering ability of DBR in vivo, but also confirm that the microbial constituents of DBR exhibit activity that influences the intestinal microbiota in a beneficial manner consistent with cholesterol lowering activity. Hence, this work provides valuable proof-of-principle support for clinical use of this specific combination of probiotics and digestive enzymes as a dietary supplement for individuals with hypercholesterolemia.

## Methods

### Daily Body Restore (DBR)

DBR consists of capsules containing a proprietary blends of probiotics (116.20 mg total weight); specifically, *Bifidobacterium infantis*, *Bifidobacterium bifidum, Lactobacillus acidophilus, Lactobacillus salivarius, Lactobacillus plantarum, Lactobacillus rhamnosus, Bifidobacterium longum, Lactobacillus casei, Lactobacillus paracasei,* and digestive enzymes (272.65 mg total weight); specifically, amylase, glucoamylase, lipase, bromelain, maltase, lactase, hemicellulose, xylanase, papain, and invertase.

### Mice

C57BL/6J mice were bred at the Malaghan Institute for Medical Research (Wellington, New Zealand) and experiments were performed at Trinity Bioactives (Wellington, New Zealand). All protocols were approved by the Animal Ethics Committee at the University of Otago, Wellington, New Zealand (Protocol AEC3-12) and animals received care in compliance with the guidelines set forth by this committee. Mice were randomly assigned to test groups consisting of 10 mice/group that were each distributed into two boxes of five animals each. All mice were first placed on a Low Fat AIN 93 M diet for 7 days. This diet has a fat content of 4.00 % (w/w) and 15.43 kJ/g caloric content. Subsequently, a control group of mice was continued on the low fat diet for the duration of the study. The other two groups of mice (Control and Experimental Groups) were fed a high fat diet beginning at week 2 through to the end of the study. This high fat diet was intended to simulate human diets enriched in fat and sugar and contained a fat content of 35.00 % and a calorie content of 22.40 kJ/G, which contained beef tallow and hydrogenated vegetable shortening as the lipid components. Mice were given the high fat diet for 13 weeks to establish hypercholesterolemia. Subsequently, the high fat diet-fed group was divided into two cohorts of mice for the experimental period. One group was supplemented with DBR in (65 μg/mL) in their drinking water while the other group was given unsupplemented tap water for the subsequent 8 weeks. To summarize, the following groups of mice were analyzed at weeks 0, 4 and 8 of the experimental period:

1. Continuation on low fat AIN 93 M Diet (n = 10)
2. High fat diet (n = 10)
3. High fat diet plus Daily Body Restore (DBR) in drinking water

### Monitoring and measurements

Animals were monitored daily while body weights were monitored once weekly throughout the trial.

Food and water consumption were monitored three times weekly. At these times, the food was replenished with fresh food and 250 mL of fresh supplemented or non-supplemented water was provided. At the time of the water change, the residual water in the bottle was recorded as the amount of water drunk.

Blood samples were taken by tail tipping at the times indicated. Blood samples were collected in tubes and stored at 4 °C overnight and the sera were aspirated and stored in fresh tubes at −20 °C. At the termination of the study, blood was also collected by cardiac puncture. Briefly, the mice were fasted for 4 h, and then anesthetized with Ketamine (100 mg/kg body weight) and Xylazine (5 mg/kg) for the cardiac puncture procedure. Sera were harvested as described.

### Serum analysis

Aliquots of serum taken from mice at time points corresponding to weeks 0, 4 and 8 of DBR supplementation or control treatment were analyzed using biochemical assay kits for Atotal cholesterol (enzymatic cholesterol assay kit; XpressBio, Frederick MD, USA), LDL (colorimetric cholesterol assay kit; XpressBio), and triglycerides (enzymatic triglycerides assay kit; XpressBio). The concentration of HDL was calculated as the difference between total cholesterol and LDL.

Cholesterol measurements were also performed using serum samples taken on weeks 0, 4 and 8 of DBR supplementation or control treatment.

### Simulator of human intestinal microbial ecosystem (Shime^®^) study

These experiments were conducted at ProDigest (Gent, Belgium). Colon reactors were inoculated with a fecal sample from a young adult donor and the experimental system was conducted as described previously [17]. Briefly, the Shime^®^ system consists of a series of double-jacketed vessels, simulating the digestive compartments. After inoculation, a two-week start up period was conducted to allow the microbial community to differentiate in the reactors. Subsequently, the reactors were run for a two-week period in which standard Shime^®^ feed was dosed to the system. The medium given consisted of arabinogalactan (1 g/L), pectin (2 g/L), xylan (1 g/L), starch (4.2 g/L), glucose (0.4 g/L), yeast extract (3 g/L), peptone (1 g/L), mucin (4 g/L), cysteine (0.5 g/L). This 2-week feeding with medium established the baseline microbial community composition and activity in the different reactors and was considered the control period. Subsequently, in the treatment period the Shime^®^ reactor was operated under nominal conditions but including supplementation with DBR for 3 weeks. Supplementation was conducted using a sinker was used to accommodate 1 capsule of DBR in the stomach/small intestine compartment.

Metabolic parameters were evaluated and compared in the Shime^®^ vessels during the 2 week control period and the three-week treatment period of supplementation with DRB. The production of short-chain fatty acids (i.e. propionate) was evaluated by standard gas-chromatographic methods from samples collected from the reactors weekly. Lactate production was measured in the reactors to evaluate microbial metabolic activity [18]. The composition of microbial communities, lactobacilli and bifidobacteria, were measured in each reactor by quantitative RT-PCR once weekly in the control and treatment periods.

### Statistical analysis

For assessing the statistical significance between groups, the following tests were performed: (1) Means and standard errors of means; (2) Assessment of statistical significance using independent Students *t* test for comparison of mean values between two populations where p < 0.05 is considered significant; and, (3) Assessment of outlier removal using Cook’s distances and z scores. Statistical analyses were conducted with and without outliers. All data are displayed as the absolute changes and averages percentages ± SEM for the indicated parameters.

## Results

### DBR significantly reduces LDL and increases HDL in a mouse model of hypercholesterolemia

Feeding a high fat diet to mice can be used to model obesity syndromes, insulin resistance, and atherosclerosis [19]. To evaluate the influence of DBR on cholesterol metabolism in vivo, mice were fed a high calorie, high fat diet and were subsequently supplemented with DBR in their drinking water to evaluate its impact on the deleterious diet. The high fat diet was provided to two groups of mice for a total of 13 weeks to establish the obesity syndrome and high cholesterol. Subsequently, the high fat diet was continued along with DBR treatment for 8 weeks in one group of mice and the high fat diet alone (unsupplemented) in the control cohort. The third group of mice was given a low fat diet for the duration of the study and used as a comparison for changes in metabolic parameters.

Monitoring of body weights over the course of the study revealed that, as anticipated, mice fed a high-fat diet gained weight at a faster rate than those receiving the standard diet. However, there was no significant difference in mean percentage of body weight gain between DBR supplement and unsupplemented mice fed a high fat diet. Specifically, the mean percentage of body weight gain during the experimental period was 4.91 ± 1.26, 15.88 1.55, 14.56 ± 1.26 % in the low fat, high fat, and high fat/DBR supplemented diet groups, respectively. Additionally, food and water consumption were not significantly different between DBR-supplemented mice and control mice given high fat and low fat diets. Mean group food consumption measurements were 492.44 ± 31.29, 579.71 ± 75.46, and 614.21 ± 50.41 g/mouse for the low fat, high fat, and high fat/DBR supplemented diet groups, respectively. Water consumption measurements were 158.51 ± 32.02 and 177.05 ± 19.04 mL/mouse in the unsupplemented and DBR-supplemented mice fed a high fat diet, respectively.

**Fig. 1:**
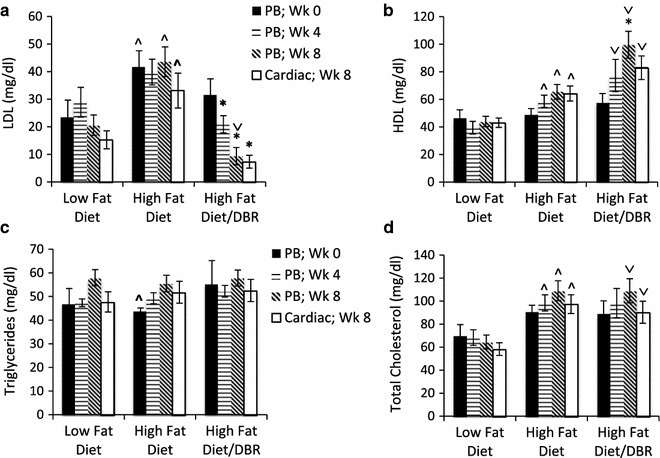
DBR supplementation reduces LDL cholesterol and increases hdl cholesterol in a mouse model of hypercholesterolemia. Three groups of mice were evaluated for cholesterol concentrations in serum obtained from peripheral blood (PB) or by cardiac puncture. The groups were as follows: (1) Control mice maintained on a low fat diet; (2) Control mice maintained on a high fat diet; and, (3) Experimental mice maintained on a high fat diet and supplemented with DBR in their drinking water. Analyses of LDL (**a**), HDL (**b**), triglycerides (**c**), and total cholesterol (**d**) were performed using serum taken at week 0, 4 and 8 of DBR or control treatments. * High fat diet vs. high fat diet + DBR is statistically significant difference (p < 0.05). ^ Low fat diet vs. high fat diet is statistically significant. ∨ Low fat diet vs. high fat diet + DBR is statistically significant

Analysis of serum cholesterol levels was performed after 4 and 8 weeks of DBR supplementation using blood samples obtained from tail tips or by cardiac puncture. The data summarizing blood cholesterol concentrations between the treatment groups are summarized in Fig. 1a. As expected, LDL, the fraction of serum cholesterol associated with atherogenesis and cardiovascular risk, was increased by a high fat diet as compared to the low fat diet. Specifically, prior to DBR supplementation (week 0), the LDL values in peripheral blood were 23.64 ± 6.01, 41.75 ± 5.84, and 31.56 ± 5.85 mg/dl in the low fat and the two high fat diet-fed groups (corresponding to unsupplement control and DBR-treated), respectively (p = 0.045 low fat vs. control high fat diet groups). The high fat diet groups therefore had LDL values that were 76.6 and 33.5 % higher than those of low fat diet-fed control mice, indicating that this dietary regimen was very effective at inducing hypercholesterolemia.

After 4 weeks of supplementation with DBR, the results revealed that this probiotic formulation had a significant impact on LDL cholesterol (Fig. 1a). In the high fat diet group that was not supplemented with DBR, LDL concentrations in peripheral blood did not significantly change between weeks 0 and 4: the values were 41.75 ± 5.84 and 39.85 ± 4.64 at these time points. However, in the DBR-treated group, LDL concentrations had dropped significantly after 4 weeks of treatment. In comparison to week 0 where high fat diet/DBR-treated mice had mean serum LDL levels of 31.56 ± 5.85 mg/dL, these levels were reduced to 20.88 ± 3.12 mg/dl after 4 weeks, amounting to a 33.8 % decrease in harmful cholesterol levels. When compared to unsupplemented mice on the high fat diet, the animals treated with DBR had LDL concentrations that were approximately half at week four 0.003 (39.85 ± 4.64 and 20.88 ± 3.12 mg/dL, respectively; p = 0.003).

Evaluation of LDL in peripheral blood after another 4 weeks of supplementation or control treatment (week 8 of the study) revealed further reductions in harmful cholesterol mediated by treatment with DBR (Fig. 1a). In mice that received the fatty diet but were not supplemented with DBR, LDL increased from 39.85 ± 4.64 to 43.60 ± 5.38 between weeks 4 and 8 of the study, indicative of the ongoing deleterious effects of the fatty diet. However, in DBR-treated mice given this same diet, a 70.3 % decrease in LDL could be appreciated by week 8 relative to the beginning of the study (9.37 ± 3.12 vs. 31.56 ± 5.85). Comparisons of DBR-treated mice and unsupplemented mice at week 8 of the study revealed a 78.5 % reduction of LDL in mice that received the probiotic supplement (9.37 ± 3.12 vs. 43.60 ± 5.38 mg/dL; p = 0.00003).

In addition to peripheral blood, LDL in serum obtained by cardiac puncture was also analyzed after eight weeks of DBR or control treatment (Fig. 1a). Mice fed the fatty diet but not treated with the probiotic had a 117 % higher concentration of serum LDL in systemic blood as compared to control mice (15.27 ± 3.25 and 33.14 ± 6.35, respectively; p = 0.03). However, LDL was very low in mice that had been treated with DBR; 7.36 mg/dL or 78 % lower than in control mice given the high fat diet without DBR (p = 0.001). In both peripheral blood and systemic blood derived from cardiac puncture, LDL levels in DBR-treated mice after 8 weeks were even lower than those in mice receiving the low fat diet (p = 0.006 and p = 0.06, respectively). Collectively, these results demonstrate that DBR affords significant and progressive reduction of harmful LDL levels in the circulation.

Assessment of HDL, which is generally correlated with cardiovascular health, was measured as the difference between total cholesterol concentrations and LDL concentrations in serum for each time point (Fig. 1b). No statistically significant difference between the three groups could be appreciated between low and high fat diet groups before DBR supplementation commenced. After 4 weeks of DBR supplementation, there was an increase in HDL in serum as compared to unsupplemented, high fat diet-fed mice although the results were not statistically significant (77.31 ± 11.65 and 58.59 ± 4.44, respectively; p = 0.14). However, after 8 weeks, comparison of DBR-treated and untreated yielded statistically significant increases in HDL in the former group. The HDL concentrations were 99.59 ± 9.77 and 65.45 ± 5.36, in probiotic supplemented and control groups, respectively (p = 0.007). In DBR-treated mice, this represented a 72.4 % increase in HDL over the course of the 8-week supplementation period (57.78 ± 6.50 vs. 99.59 ± 9.77). Moreover, the HDL levels in serum of DBR-treated mice were dramatically increased (127 %) relative to those in control mice given the low fat diet for the duration of the study (99.57 ± 9.77 vs. 43.93 ± 3.73). In systemic blood obtained by cardiac puncture, HDL was also significantly increased by DBR treatment (Fig. 1b). Mice given the probiotic had HDL cholesterol concentrations that were 29.1 % higher than those given the fatty diet alone (82.98 ± 8.55 and 64.27 ± 5.46, p = 0.07).

We also investigated the influence of DBR on triglycerides, which serve as biomarkers of cardiovascular risk, in mice fed a high fat diet (Fig. 1c). The data showed that the high fat diet did not significantly alter triglyceride levels in this mouse model as compared to mice fed a low fat diet. After 4 and 8 weeks of DBR supplementation, there were no significant differences in triglyceride concentrations relative to the unsupplemented control group.

Lastly, total serum cholesterol was compared between the three cohorts of mice at weeks 0, 4 and 8 of DBR or control treatment (Fig. 1d). Expectedly, mice fed the low fat diet had significantly lower serum cholesterol levels as compared to those given the high fat diet. However, comparison of DBR-treated vs. unsupplemented mice on the high fat diet did not reveal significant differences in total cholesterol in either peripheral or systemic blood.

Collectively, these data from the mouse model of hypercholesterolmemia demonstrate that treatment with the DBR supplement containing a proprietary blend of probiotics and digestive enzymes specifically reduces harmful LDL in serum while increasing HDL.

### DBR supplementation increases metabolic activity of microbiota in the shime^®^ system

In this study, we first evaluated the impact of DBR on fermentation metabolites in the Shime^®^ model where the interactions of this supplement with native microbiota corresponding to conditions mimicking the ascending, transverse and descending colon were tested. The concentrations of short chain fatty acids (SCFA) produced in the Shime^®^ reactors were measured. SCFA are produced in the gut from fermentation of indigestible carbohydrates including dietary fiber, resistant starch, and oligosaccharides, and are absorbed from the colon into the liver as well as peripheral tissues. Production of SCFA is beneficial to numerous aspects of the digestive process including stimulation of the immune system [20] and protection of the colon against cancer [21, 22]. Propionate in particular has been noted to decrease cholesterol synthesis in the liver, improving lipid metabolism [23]. Since some strains of *Bifidobacterium* and *Lactobacillus* have been associated with improved cholesterol profiles and overall gut health [24–30], we tested the influence of DBR on simulated small intestine conditions.

**Fig. 2:**
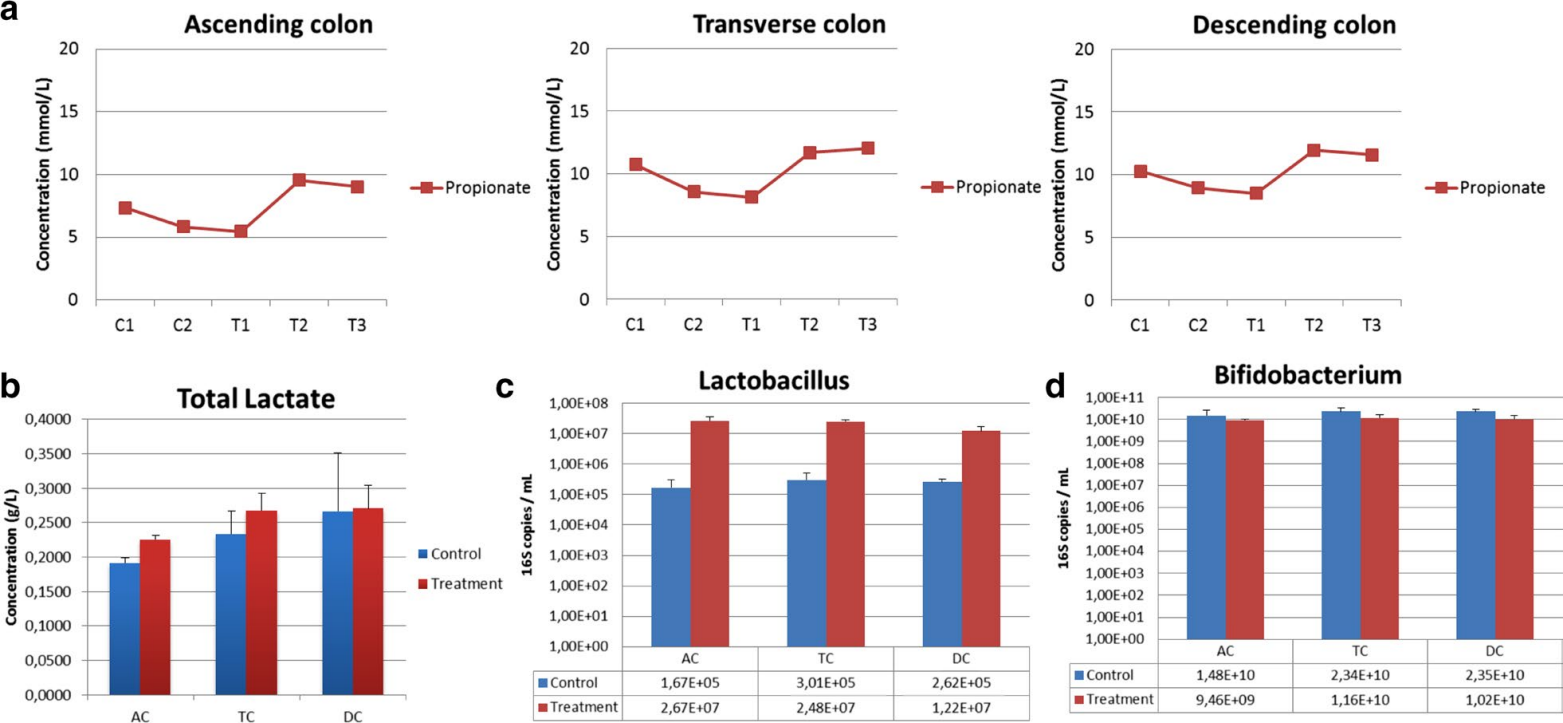
Analysis of DBR in the Shime^®^ model. **a** Propionate production in the ascending and transverse colon in DBR-supplemented vessels (treatment weeks* T1*,* T2* and* T3*) vs. control vessels (*C1* and* C2*). **b** Total lactate concentrations (g/L) in DBR supplemented treatment vessels vs. controls. **c**, **d** Quantitative PCR results for the total copies/mL of lactobacilli (**c**) and bifidobacteria (**d**)

Our results showed that addition of DBR significantly improved propionate production in the vessels corresponding to the ascending colon and transverse colon during the 3 week supplementation period based on the comparison to the control (non-supplemented) two-week time period (Fig. 2a). Accordingly, an increased production of the metabolite lactate, a by-product of the fermentation process, could be appreciated in the ascending and transverse colon reactors (Fig. 2b). Moreover, the presence of lactobacilli was also increased in all the Shime^®^ reactor vessels (Fig. 2c), although no change in the *Bifidobacteria* population could be appreciated in the DBR-supplemented system vs. the non-supplemented control vessels (Fig. 2d). Together, these results are suggestive of survival and/or activity of probiotic bacteria from DBR under conditions set up to mimic the human gastrointestinal tract. The improved propionate production in DBR-supplemented vessels corresponding to the proximal colon is also suggestive of the potential of DBR as a modulator of de novo fatty acid and cholesterol synthesis.

## Discussion

In this study, Daily Body Restore (DBR), a proprietary blend of *Lactobacillus* and *Bifidobacteria*, and digestive enzymes, was evaluated in vitro and in an animal model to gauge its candidacy as a clinical cholesterol-lowering product. The data generated using these two model systems support the conclusion that the probiotic strains in DBR are metabolically active and can modulate cholesterol metabolism in a favorable manner. In a mouse model intended to simulate high fat, high sugar diets, DBR treatment afforded significant reductions in harmful LDL and improvements in HDL, even improving cholesterol profiles relative to those of control mice fed normal low fat diets. What is more, in the Shime^®^ model that simulates the compartments of the gastrointestinal tract, addition of DBR to the vessels had a positive impact on the activity of intestinal microbiota. Microbial fermentation was increased by DBR, as evident by increased lactate acid production in the reactors and an increase in lactobacilli. Our results did not show a change in the total load of Bifidobacteria in the DBR-treated vs. control reactors; however, this finding does not rule out the possibility that the composition of Bifidobacteria species and/or their metabolic activities, including lipid-lowering effects, may have been modulated by DBR treatment. In addition to probiotics, DBR also contains digestive enzymes that likely contribute to increased nutrient absorption in vivo, and may also improve cholesterol management; specifically, lipase, a central enzyme in lipid metabolism.

Moreover, evaluation of short chain fatty acid production in the Shime^®^ reactors revealed that propionate production was increased in the ascending colon and transverse colon vessels during treatment with DBR. This fermentation by-product of carbohydrate digestion is established as having cholesterol-lowering activity [23]. Hence, the Shime^®^ model demonstrated the stability and activity of DBR in a dynamic ecosystem. Clinical studies are underway to investigate the impact of DBR in individuals afflicted with hypercholesterolemia and/or having cardiovascular risk factors.

In recent years, much attention has been given to the ability of probiotics to reduce lipids and cholesterol levels with evidence supporting several mechanisms of action. Specific bacteria, including specific *Lactobacillus* strains [31], have been reported to assimilate cholesterol in vitro, thereby presumably lowering luminal cholesterol levels available for absorption in vivo [32–34]. Moreover, *Lactobacillus* bacteria can produce ferulic acid (FA), which can inhibit hepatic HMG-CoA reductase [35, 36], a target of statin drugs. Another proposed mechanism of action of lactobacilli and bifidobacteria for lowering serum cholesterol is deconjugation of bile acids and increasing their rate of excretion, which may increase the demand for de novo bile acid synthesis from cholesterol [30, 37]. Notably, lactobacilli exhibit strain-specific differences in enzymatic activity of bile-salt hydrolase and therefore have disparate propensities to remove cholesterol from culture medium and to lower serum LDL-C and triglycerides in animals [37]. It is important to note that not all lactic acid bacteria, combinations of strains, or specific formulations can be expected to enact a particular cholesterol-lowering mechanism [6]. For this reason, individual formulations should undergo testing to look for metabolic activity and stability of the probiotic, as has been performed in the present study for our proprietary formulation.

Animal studies have routinely been employed to investigate the effects of prescription drugs and herbal remedies/natural products as cholesterol modifiers. For example, in a mouse model of atherosclerosis, resveratrol (a natural phenol and phytoalexin) decreased plasma levels of LDL by 19 %, comparable to atorvastatin (Lipitor^®^) alone (19 %), and the combination of both agents decreased LDL by 22 % over the 14-week study period [38]. In another example of an experimental study, LDL levels were reduced from 53 % to 67 % by ezetimibe (a cholesterol absorption inhibitor) treatment in a mouse model [39]. Direct comparisons of the present results to other animal studies are precluded by the vast variability in experimental parameters between studies, including the specific animal model, the duration of treatment, and the time points of cholesterol measurement. However, when placed broadly into the context of existing experimental data in conjunction with our data from the Shime^®^ study, we conclude that DBR appears to be remarkably potent and is therefore a promising candidate for clinical studies.

Our observations that DBR increases the concentrations of propionate in the vessels corresponding to the ascending and transverse colon in the Shime^®^ system are suggestive of a potent effect of this probiotic supplement on cholesterol metabolism. Of the short chain fatty acids that are typically monitored in the Shime^®^ studies (which also include acetate and butyrate), propionate is specifically associated with beneficial effects on both lipid and glucose metabolism [40, 41]. Short-chain fatty acids such as propionate are produced by fermentation by intestinal microbes and are readily absorbed from the large bowel into the liver in vivo where cholesterol synthesis can be inhibited [5]. The concentrations of propionate, which are influenced by the fermentation substrate and the microbial composition, can serve as markers for the effects of DBR on cholesterol. High propionate levels reportedly lead to a competitive inhibition of the activity of acetyl-coenzyme A synthetase involved in cholesterol synthesis [42, 43]. Studies where oral propionate was administered to animals fed hypercholesterolemic diets high in saturated fat revealed lowering of liver cholesterol levels [23, 44]. Decreased total concentrations of cholesterol in plasma have also been reported in animals [45, 46] as well as increased beneficial HDL in humans [47] given propionate orally. Interestingly, one study revealed that rectal administration of propionate to animals had a much more potent cholesterol-lowering effect compared with dietary supplementation, suggesting that propionate in the colon is a highly potent modulator of cholesterol metabolism [23]. Therefore, probiotics such as DBR that possess the ability to increase propionate production are expected to be associated with lowering of serum cholesterol and possibly reducing cardiovascular disease risk.

## Conclusions

This report provide proof-of-concept for the cholesterol-lowering effects of DBR, a blend of 9 probiotic organisms of the genera *Lactobacillus* and *Bifidobacterium*, and 10 digestive enzymes, thereby providing a promising basis for future clinical studies. Specifically, the probiotic constitution supplied by DBR capsules is demonstrated to be active in a simulated intestinal microenvironment, and can lower harmful LDL while raising HDL levels in experimental animals.
